# Structural and Gelation Characteristics of Alkali-Soluble β-Glucan from *Poria cocos*

**DOI:** 10.3390/gels11060387

**Published:** 2025-05-24

**Authors:** Zhixing Li, Chenglei Sun, Fan Wang, Zhaofei Xia

**Affiliations:** 1 College of Veterinary Medicine, China Agricultural University, Beijing 100193, China; lzx1333@126.com (Z.L.); wangfan1112024@163.com (F.W.); 2 College of Agricultural and Life Sciences, The University of Tokyo, Tokyo 113-8654, Japan; sunclson@163.com

**Keywords:** *Poria cocos*, alkali-soluble polysaccharide, β-glucan, hydrogel

## Abstract

Alkali-soluble polysaccharides from *Poria cocos* (APCP) are typically discarded due to poor water solubility and limited bioavailability, despite their β-(1→3)-glucan backbone suggesting potential for functional applications. This study aimed to explore the structural characteristics, gelation behavior, and the capacity of APCP to reduce silver ions. Structural analysis confirmed that APCP is a homogenous β-(1→3)-D-glucan with a molecular weight of 314.2 kDa and a PDI of 1.32. A pH-mediated strategy enabled the formation of stable single-component APCP hydrogel (APCPH) with tunable mechanical strength, high swelling capacity (>590%), and thermal stability. The APCPH further acted as both a reducing and stabilizing matrix for in situ AgNP formation. Notably, the Ag-APCP hydrogel exhibited distinct antibacterial activity, with inhibition zones reaching 5.31 mm against *Staphylococcus pseudintermedius*. These findings demonstrate the feasibility of transforming underutilized APCP into multifunctional hydrogel platforms for green nanomaterial synthesis and biomedical applications. Future studies will focus on optimizing AgNP synthesis parameters and evaluating long-term stability and biocompatibility for translational use in antimicrobial therapies.

## 1. Introduction

Hydrogels are three-dimensional networks formed via physical or chemical crosslinking [1]. Although synthetic hydrogels offer tunable mechanics and programmable functions, their biomedical and food-industry applications remain hampered by their limited biocompatibility, uncontrolled degradability, and reliance on nonrenewable feedstocks [2]. Natural polysaccharides have emerged as promising materials for hydrogels due to their intrinsic biocompatibility, renewability, and abundance in nature [2,3,4]. β-glucans are natural polysaccharides of D-glucose units joined by β-glycosidic bonds. They occur abundantly in fungal and yeast cell walls, marine algae, and cereals [5]. Variations in molecular weight, primary structure, and conformation among β-glucans underlie their distinct physicochemical and bioactive properties [6,7]. Although β-glucans exhibit unique sol–gel and self-assembly behaviors, studies of single-component β-glucan hydrogels remain underexplored due to their poor mechanical strength and limited solubility. Consequently, most research has focused on hybrid systems [8]. For example, the addition of oat β-glucans enhances the viscoelasticity and thermal stability of konjac glucomannan gels [9]. Hyperbranched β-glucans extracted from Pleurotus tuber-regium combined with xanthan gum forms a self-healing hydrogel upon crosslinking [10]. To fully exploit β-glucan resources, correlational research on structural characterization and gelation is required.

*Poria cocos*, a brown-rot fungus of the Polyporaceae family, primarily colonizes the roots of *pinus* species [11]. First documented in the ancient Chinese pharmacopeia Shen Nong Ben Cao Jing (The Divine Farmer’s Materia Medica), it was historically classified as a “Superior Class” herb. Pharmacological studies have confirmed its nephroprotective, spleen-tonifying, and neurosedative activities [12]. Modern pharmacological studies have identified more than 190 active compounds in *Poria cocos*, among which polysaccharides are considered the principal bioactive components, accounting for 70–90% of the dry weight of the sclerotium [13]. Poria cocos polysaccharides (PCP) comprise two structural classes: β-glucans with β-(1→3)-linked backbones and β-(1→6)-side chains, and heteropolysaccharides containing methylpentoses, pentoses, mannose, and galactose [13,14,15]. Based on solubility, PCP are divided into water-soluble polysaccharides (WPCP) and alkali-soluble polysaccharides (APCP), which account for the majority (>80%) of PCP [16]. APCP is typically extracted following hot-water removal of the water-soluble fraction, using alkaline solutions—most commonly sodium hydroxide (NaOH)—to solubilize the residual polysaccharides. The extract is then neutralized and precipitated with ethanol to obtain crude APCP. Recent studies have also explored advanced extraction techniques such as ultrasound-assisted and deep eutectic solvent (DES)-based methods to improve yield and preserve structural integrity [16,17]. Despite these advances, APCP remains far less studied than WPCP. Due to poor water solubility, APCP has historically been discarded as extraction residues, leading to significant resource underutilization and environmental burden [17,18]. Moreover, the functional properties and structure–activity relationships remain poorly defined, which further limits application potential of APCP. Accordingly, strategies to valorize these underused polysaccharides into functional materials are urgently needed.

Among various metallic nanoparticles, silver nanoparticles (AgNPs) are particularly attractive due to broad-spectrum antimicrobial activity and compatibility with green synthesis routes. AgNP synthesis encompasses three primary strategies: physical, chemical, and biological methods [19]. While physical and chemical methods dominate industrial-scale production, biosynthetic approaches have attracted scholarly interest. Biological synthesis utilizes microorganisms or plant metabolites to reduce Ag^+^ [20,21,22], achieving eco-friendly production with biocompatibility, making it ideal for biomedical applications. Fungal polysaccharides, particularly β-glucans, provide a natural, non-toxic, and biocompatible matrix that has been widely used in hydrogel formation and nanomaterial stabilization [23]. Numerous studies have demonstrated that fungal β-glucans can mediate the green synthesis of AgNPs. β-glucan from *Hypsizygus marmoreus* has been shown to synthesize cytotoxicity-free AgNPs [24]. Schizophyllan (linear β-1,3-glucan) enables the biosynthesis of spherical AgNPs [25]. APCP has been reported to consist primarily of (1→3)-linked glucose residues, structurally analogous to curdlan and other functional β-glucans [26,27]. Given the β-glucan-rich structure of APCP, we hypothesized that it possesses inherent gel-forming ability and can be utilized as a functional matrix for green AgNP synthesis, thereby enhancing the practical value of this underused polysaccharide.

To test this hypothesis, we systematically investigated the structural characteristics, gelation behavior, and Ag^+^ reduction capability of APCP. A simple, green, and safe method was developed to produce a stable single-component hydrogel. The effects of pH and polysaccharide concentration on gelation were evaluated, along with the hydrogel’s rheological properties, microstructure, and thermal stability. Additionally, the swelling behavior, water retention, and antioxidant activity of the hydrogel were evaluated. The APCPH was capable of reducing Ag^+^ to AgNPs. To our knowledge, this is the first report of the inhibitory effect of this APCPH against *Staphylococcus pseudintermedius*, marking a significant advance in the development of antimicrobial hydrogels. These findings support the potential of APCP as a multifunctional material for biomedical hydrogel applications and expand the current understanding of β-glucan-based gelation systems.

## 2. Result and Discussion

### 2.1. Structural Characterization of APCP

#### 2.1.1. Monosaccharide Composition and Molecular Weight Determination of APCP

**Figure 1:**
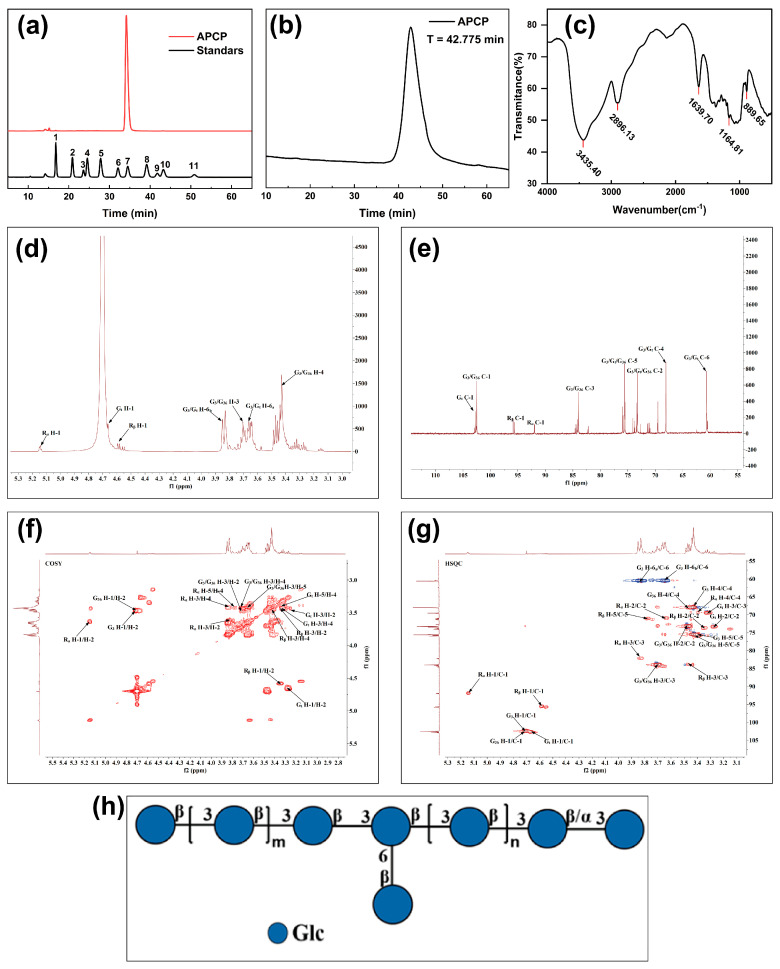
(**a**) HPLC chromatogram of monosaccharide in the polysaccharide fraction of complex standard monosaccharides and the APCP (1: Man; 2: GlcN; 3: Rha; 4: GlcA; 5: GalA; 6: GalN; 7: Glc; 8: Gal; 9: Xyl; 10: Ara; and 11: Fuc); (**b**) HPGPC chromatogram of the APCP; (**c**) the FT-IR spectrum of the APCP; (**d**–**g**) the ^1^H NMR, ^13^C NMR and COSY, HSQC spectra of the APCP; and (**h**) the possible chemical structure of the APCP.

Under the same experimental conditions, the monosaccharide composition of the APCP was determined by comparing its retention times with those of monosaccharide standards. As shown in Figure 1a and Table 1, the results revealed that the APCP mainly consisted of glucose (99.896%) with traces of mannose (0.104%). The glucose-dominated monosaccharide profile was consistent with the FT-IR data, which indicated the predominant presence of pyranose rings in the APCP. To better understand the homogeneity and molecular weight distribution of the APCP, HPGPC was employed. As shown in Figure 1b, a symmetrical single peak was observed, demonstrating good homogeneity. Molecular weight parameters were quantified as follows: peak molecular weight (Mp) was 282.8 kDa, weight–average molecular weight (Mw) was 314.2 kDa, and number–average molecular weight (Mn) was 237.4 kDa. The polydispersity index (PDI) was the ratio of Mw to Mn. The results showed that the PDI of the APCP was 1.32, which was significantly lower than the standard threshold for homogeneous biomacromolecules (PDI < 1.5) [28]. This indicated that the molecular weight distribution of the polysaccharide was narrow. The molecular chain length was highly consistent. These features were characteristic of monodisperse polysaccharides, meeting the needs of subsequent research. Prior investigations characterizing alkali-extracted polysaccharides from Poria cocos reported approximating 1.995 and 1.632 in samples CMP1 and CMP3 [16], respectively, which were both higher than the PDI of the APCP obtained in this study. Polysaccharides with low PDI generally exhibit more consistent physicochemical properties and enhanced biological activity [29]. This phenomenon was closely related to the integrity of the molecular structure of polysaccharides during the extraction process, suggesting that the extraction process used in this study caused relatively less damage to the polysaccharide chains.

#### 2.1.2. The Glycosidic Bond of the APCP

To investigate the functional group structure of the APCP, FT-IR spectroscopy was employed. As shown in Figure 1c, several characteristic absorption peaks were observed. Peaks at 3435.40, 2896.13, 1639.70, and 1164.81 cm^−1^ corresponded to O–H stretching, C–H stretching, C=O stretching, and C–O–C asymmetric stretching vibrations, respectively [30]. The absorption at 891 cm^−1^ confirmed the β-configuration of the pyranose ring. Moreover, strong absorption bands between 1150–1010 cm^−1^, particularly at 1081.03 cm^−1^ and 1035.17 cm^−1^, further supported the presence of pyranoside moieties. The characteristic β-pyranoside linkage was identified at 889.65 cm^−1^ [31]. These results together indicate that the APCP contain β-glycosidic bonds.

To determine the glycosidic linkage types, partially methylated alditol acetates (PMAAs) derived from APCP through methylation, hydrolysis, and acetylation were analyzed by GC-MS. Five methylated fragments were detected (Table 2), corresponding to terminal-Glcp (t-Glcp, 12.272%), 3-Glcp (77.497%), 3,4-Glcp (1.244%), 2,3-Glcp (4.018%), and 3,6-Glcp (4.970%). The dominance of 3-Glcp linkages (77.497%) was consistent with the monosaccharide analysis, which showed 0.104% mannose in the APCP. Moreover, no mannose-derived glycosidic linkages were detected after methylation. Collectively, these findings suggest that the APCP features a backbone primarily composed of (1→3)-linked glucopyranosyl units.

To verify the chemical structure of APCP, 1D and 2D NMR analyses were performed (Figure 1d–g and Figure S1a–d). In the HSQC and COSY spectra, five anomeric H/C coupling signals were detected: δ 4.71/102.46 ppm (G_3_), δ 4.72/102.37 ppm (G_36_), δ 4.67/102.62 ppm (G_t_), δ 5.14/91.79 ppm (R_α_), and δ 4.59/95.65 ppm (R_β_). Following the assignment of anomeric signals, the ^1^H and ^13^C chemical shifts of saccharide residues were resolved through multidimensional NMR analyses (HSQC, COSY, HMBC, and NOESY), with spectral interpretations systematically corroborated by ^1^H/^13^C NMR datasets and alignment to literature references (Table 3) [31,32,33,34,35]. G_3_ and G_36_ corresponded to →3)-β-D-Glcp-(1→, while Gt represented β-D-Glcp-(1→. The anomeric signal (δ 5.14/91.79 ppm) verified the α-configuration of R_α_ (→3)-α-D-Glcp), whereas R_β_ (→3)-β-D-Glcp) exhibited characteristic β-linkage features.

Structural analysis revealed a homogeneous β-(1→3)-D-glucopyranosyl backbone with C-6 branching which is similar to that reported by Jin [36]. This is in accordance with reported APCP structure, which predominantly possess β-pyranose configurations. Conversely, α-glucans are generally water-soluble. Fungal β-glucans typically possess β-(1→3)-linked backbones (>50% abundance) with β-(1→6) or β-(1→2) branches. Notably, β-(1→3) linkages are essential for bioactivity, as demonstrated by Chihara et al., who observed enhanced suppression of S180 sarcoma in β-(1→3)-D-glucans following the removal of β-(1→6) side–chains [37]. Similar structural patterns were observed in other fungal polysaccharides, as seen in the *Pholiota nameko* polysaccharide with a →3)-β-D-Glcp-(1→ backbone and →6)-β-D-Glcp-(1→ branches [38].

### 2.2. Preparation and Gelation Conditions of APCP Hydrogel

Given the non-fluidity of the hydrogel, this experiment determined the formation of the hydrogel via the inversion method. As shown in Figure S2a, freeze-dried APCP appeared as a fluffy white solid. Upon dissolution in alkaline solution, it formed a slightly yellow and viscous liquid. Following pH adjustment, the APCP underwent gelation, resulting in an opaque, homogeneous, and white hydrogel. Figure S2b–d illustrated the influence of the pH value and APCP concentration on the gel behavior. As shown in Figure S2b, at a pH of 5.7, APCP within the concentration range of 1–3 wt% exhibited fast-gel characteristics (≤3 s). As illustrated in Figure S2c, with a fixed APCP concentration of 1 wt%, the hydrogel maintained rapid gelation ability (≤11 s) within the pH range from 4.7 to 6.4. It was noteworthy that the APCP demonstrated a wide pH response range (3.3–8.3). However, when the pH > 8.3, even when the concentration was increased to 4 wt%, it took a long time to form a gel (Figure S2d). The minimum gelling concentration of the APCP was 0.375 wt%. Under identical pH conditions, the gelation time was inversely correlated with the APCP concentration. In summary, the APCP could rapidly gel in a weak acidic environment (pH 4.7–6.4) and within the concentration range of 1–2.5 wt%.

### 2.3. Characterization of APCP Hydrogel

#### 2.3.1. Rheology Analysis

**Figure 2:**
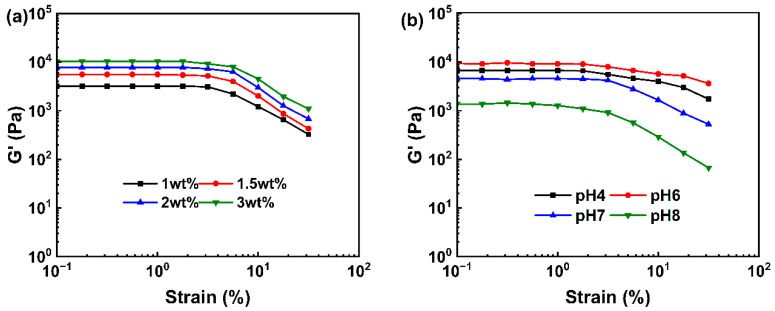
Strain-sweep rheological measurements of APCP hydrogels at 25 °C. (**a**) Variation of storage modulus (G′) with strain for hydrogels containing 1 wt% (■), 1.5 wt% (●), 2 wt% (▲), and 3 wt% (▼) APCP at pH 7.0. (**b**) Variation of G′ with strain for 2 wt% APCP hydrogels at pH 4 (■), pH 6 (●), pH 7 (▲), and pH 8 (▼).

The linear viscoelastic region (LVR) of the samples was determined via strain scanning. G′ exhibited platform characteristics within the strain range of 0.1–1% (Figure 2a), which implied that the hydrogel network structure was not significantly disrupted within this deformation range. As the strain amplitude increased, G′ gradually decreased in all samples. This indicated that excessive strain disrupted the internal cross-linked structure, causing the material to transition from a linear to a nonlinear viscoelastic state. As the polysaccharide concentration increased, the storage modulus of APCPH increased as well. This increase indicated that a higher polysaccharide concentration enhanced the gel’s resistance to deformation by increasing the cross-linking density [39]. The increase in mass fraction resulted in greater molecular chain density, promoting the formation of more cross-linking points and junction zones, thereby enhancing the gel’s mechanical stability [40]. Figure 2b illustrates the effect of different pH values on the LVR of a 2 wt% hydrogel. Upon an increase in the strain amplitude, G′ exhibited a decreasing trend. Notably, when the system pH was 6, the G′ of the APCPH reached its maximum, followed by the G′ value of the sample at pH 4. Under neutral or alkaline conditions, the G′ decreased significantly. Furthermore, the yield stress of the gel under acidic conditions increased substantially and the LVR range widened. These data suggested that a weakly acidic environment was more conducive to constructing the three-dimensional cross-linking network of the APCP gel, while neutral or alkaline conditions might lead to network loosening due to increased electrostatic repulsion between molecular chains [41,42].

**Figure 3:**
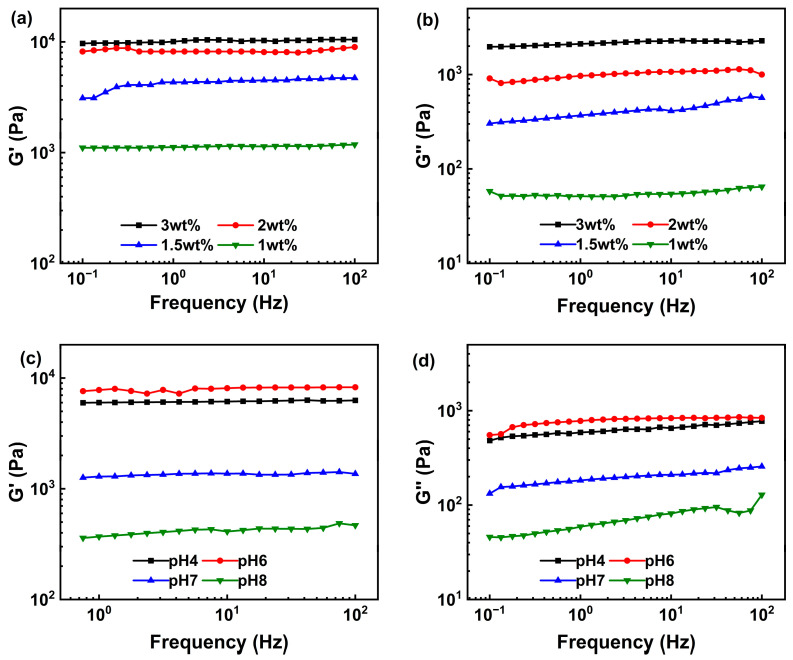
Frequency-sweep rheological measurements of APCP hydrogels at 25 °C. Variation of storage modulus (G′) (**a**) and loss modulus (G”) (**b**) with frequency for APCP hydrogels containing 1 wt% (■), 1.5 wt% (●), 2 wt% (▲), and 3 wt% (▼) APCP at pH 7; Variation of G′ (**c**) and G” (**d**) with frequency for 2 wt% APCP hydrogels at pH 4 (■), pH 6 (●), pH 7 (▲), and pH 8 (▼).

To further understand the gel’s structure and mechanical properties, a frequency sweep was performed within the LVR (1%). In the range of 0.1–100 Hz, the storage modulus of each group was significantly higher than the loss modulus. Both G′ and G″ increased depending on the concentration. The strength of the gel network was positively related to the polysaccharide concentration (Figure 3a), which was in agreement with the results of strain scanning. The effect of different pH values on the viscoelastic behavior of the APCPHs (Figure 3c,d) was also consistent with the strain sweep results. Collectively, these findings confirm that a weakly acidic environment facilitates the formation of a stable three-dimensional network in APCP gels.

#### 2.3.2. Differential Scanning Calorimetry (DSC)

**Figure 4:**
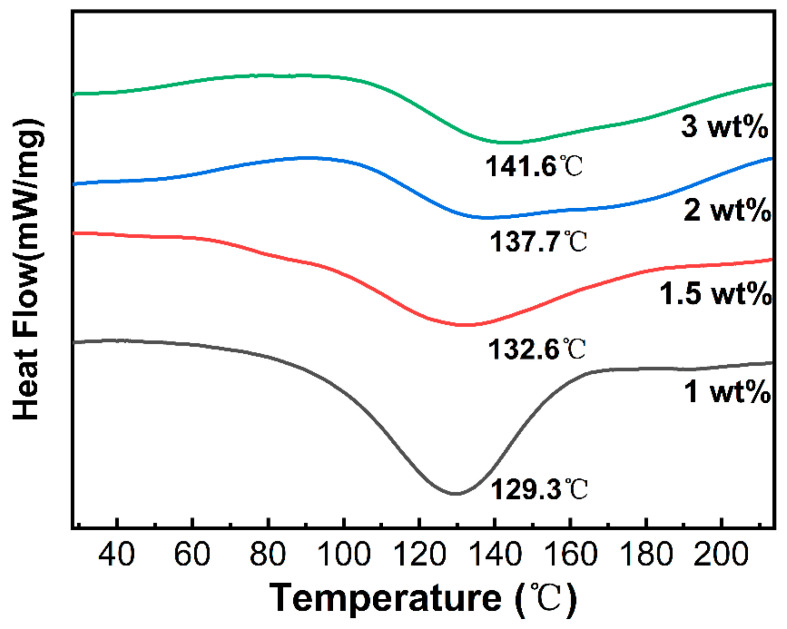
DSC patterns of the lyophilized APCP hydrogels.

The thermal stability was assessed by monitoring the hydrogel’s decomposition temperature or endothermic peak during heating. As shown in Figure 4, APCP hydrogels with varying mass fractions showed a broad endothermic peak in the 100–150 °C range. This peak corresponded to the thermal decomposition of polysaccharide components. Such decomposition involved hydrogen bond breakage, conformational changes among glucan molecular chains, side-chain decarboxylation reactions, and the thermal degradation of the sugar-ring carbon skeleton [43]. When the polysaccharide concentration rose from 1 wt% to 3 wt%, the endothermic peak temperature rose significantly from 129.3 °C to 141.6 °C. Concurrently, the enthalpy increased as the concentration went up, indicating that more energy was needed to disrupt the gel network and thereby destroy the gel structure [44]. Higher-concentration polysaccharides formed a more compact three-dimensional network. They achieved this by increasing the density of hydrogen bonds between molecular chains, strengthening the intermolecular forces, and modifying molecular interactions [43,45]. As a result, higher temperatures were required to break the intermolecular forces within the composite gel.

#### 2.3.3. Microstructure

**Figure 5:**
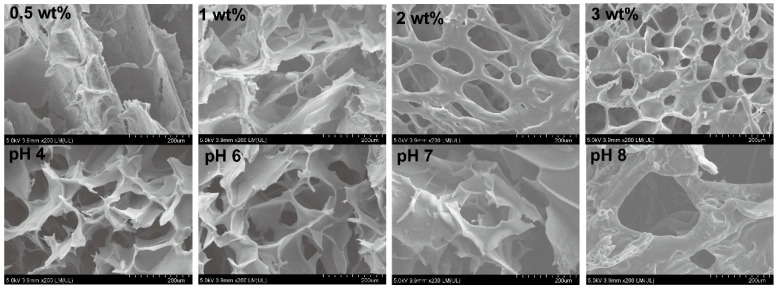
SEM images of the freeze-dried APCPH specimens.

Figure 5 shows the network structure of the APCPH. The APCPH exhibited a three-dimensional network structure comprising continuous irregular polygonal pores. The pore walls were smooth and showed a certain degree of thickness. At a polysaccharide concentration of 0.5 wt%, the APCPH demonstrated disorderly pore distribution and loose network connection. As the polysaccharide concentration increased, the gel’s network structure gradually shifted from loose to dense. Simultaneously, more heterogeneous porous structures emerged. The cellular porous morphology became more distinct, and the gel wall thickness increased correspondingly. This porous structure endowed the material with a larger specific surface area and pore volume, facilitating material loading and transfer [46]. However, an overly ordered and densely-packed hydrogel network may hinder water molecule penetration and diffusion. This may lead to a decline in swelling performance and a delay in the release rate of bioactive substances [47].

Furthermore, as the pH value increased, the structural orderliness decreased significantly. Despite these changes, the hydrogel still retained its loose and porous characteristics.

### 2.4. Molecular Forces

The formation of single-component polysaccharide gel networks depends upon weak non-covalent interactions, including hydrophobic interactions, electrostatic forces, and hydrogen bonding [48]. Given that these non-covalent interactions are the key factors in the formation of such gel networks, to understand the formation mechanism of APCPHs dissociation reagents, including NaCl, urea, and SDS, were added to 2 wt% APCPHs for validation. NaCl attenuates electrostatic interactions through charge shielding effects [49]. Urea acts as a bifunctional modulator of hydrogen bonds which can competitively displace or bridge the hydrogen bonds between polysaccharide molecules [50]. In addition, SDS disrupts intermolecular hydrophobic interactions [51].

As shown in Figure S3, the addition of SDS and urea caused progressive disruption of the hydrogel network, transitioning from a solid-like gel to a semi-solid or liquid state with increasing concentration. In contrast, samples treated with NaCl maintained typical gel characteristics across all tested concentrations, indicating a lesser impact on network integrity. These visual changes qualitatively suggest that hydrogen bonding and hydrophobic interactions are critical to APCPH network stability, while electrostatic forces play a more supportive role.

To further quantify the effect of NaCl, frequency sweep rheological analyses were performed on APCPH samples containing 0.05–0.3 M NaCl at pH 6 (Figure S4). The values of G′ and G″ remained relatively stable at NaCl concentrations up to 0.2 M, indicating that moderate ionic strength does not significantly disrupt the gel network. However, at 0.3 M NaCl, both G′ and G″ decreased markedly, suggesting that excess Na^+^ induced charge shielding effects that interfered with electrostatic coordination and disrupted the hydrogen-bonded network [50]. These findings support the conclusion that hydrogen bonding and hydrophobic interactions are the dominant stabilizing forces in APCP hydrogels, with electrostatic interactions contributing secondarily.

### 2.5. Macroscopic and Microscopic Evaluation of Ag-APCP Hydrogels

**Figure 6:**
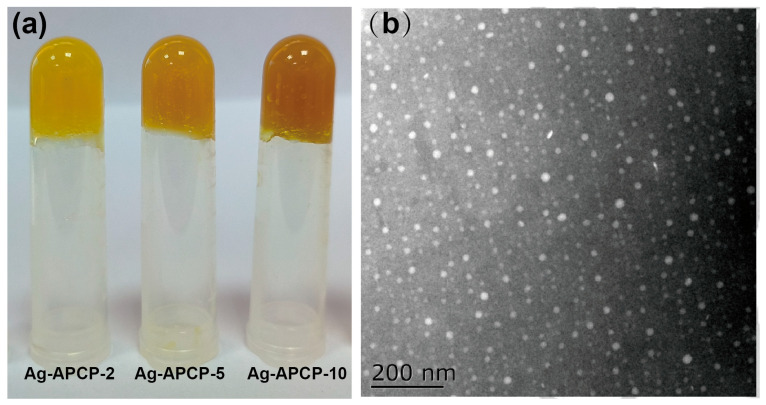
Macroscopic (**a**) and TEM image (**b**) of Ag-APCP hydrogels.

As shown in Figure 6a, the color of the Ag-APCP hydrogels progressively deepened from light yellow to dark brown with increasing AgNO_3_ concentrations (2, 5, and 10 mmol/L), suggesting the in situ formation of AgNPs. This macroscopic color change is attributable to the surface plasmon resonance (SPR) phenomenon, a hallmark optical feature of AgNPs, typically characterized by absorption in the 400–450 nm range, depending on particle size and morphology [52]. In contrast, AgNO_3_ is colorless in aqueous solutions; thus, the observed color transition further implies that the APCP acted as a reductant, facilitating the reduction of Ag^+^ to elemental silver (Ag^0^). Such reductive behavior is commonly reported in polysaccharides bearing hydroxyl and aldehyde groups, which can donate electrons during the redox reaction [53].

To confirm the formation and dispersion state of AgNPs, transmission electron microscopy (TEM) analysis was performed on the Ag-APCP-5 hydrogel (Figure 6b). The image revealed the presence of well-dispersed, spherical AgNPs with uniform size distribution and no apparent aggregation, indicating successful stabilization by the polysaccharide matrix. These observations suggest that the APCP may have not only reduced Ag^+^ but also functioned as a capping and stabilizing agent, thereby potentially preventing nanoparticle agglomeration. Such dual functionality has been similarly proposed in other natural polymer-based nanocomposite systems [54,55,56].

### 2.6. Swelling Ratios and Water-Holding Capacities

**Figure 7:**
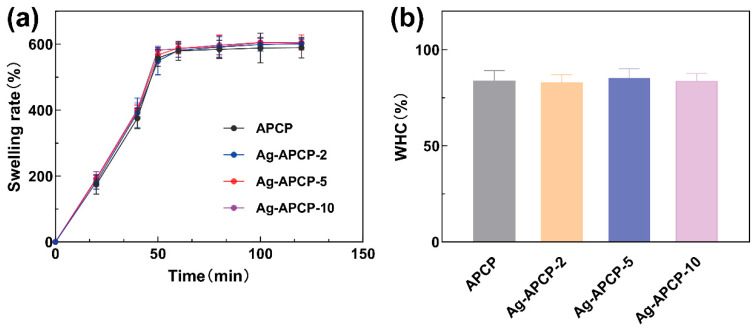
Water retention and swelling properties of APCP hydrogels and Ag-APCP hydrogels. (**a**) swelling rate; (**b**) WHC.

The swelling ratio and water-holding capacity (WHC) are fundamental indicators for evaluating hydrogel performance, particularly in biomedical applications such as wound dressings. The swelling behavior reflects a hydrogel’s ability to absorb and retain large volumes of water or biological fluids, which is crucial for maintaining a moist wound environment and facilitating tissue regeneration [57]. Hydrogels exhibit a three-dimensional porous network capable of entrapping water molecules via capillary action and hydrogen bonding [4]. As shown in Figure 7a, the hydrogels in each group attained swelling equilibrium within 60 min, with a swelling ratio exceeding 590%. This suggests that the hydrogel possesses sufficient swelling capacity to absorb exudate and establish a moist healing environment for wounds. WHC, defined as the ability of a hydrogel to retain absorbed water under external stress or gravitational force, is another critical performance metric. As illustrated in Figure 7b, the WHC values of all samples were above 81%, confirming their strong water-retention capability, which is essential for prolonged hydration and sustained drug release in biomedical contexts. Moreover, the incorporation of AgNO_3_ at varying concentrations (2–10 mmol/L) did not significantly affect either the swelling ratio or WHC of the hydrogels. This suggests that the loading process of AgNPs did not compromise the physical integrity or porous nature of the APCP matrix. These results were consistent with the findings of Jiang et al., who reported that the number of pores, rather than the concentration of Ag, primarily governs the hydrophilicity and water absorption of AgNP-containing hydrogels [58].

### 2.7. Antioxidant Activities

APCP was known to exhibit significant antioxidant activity, primarily attributed to its abundant hydroxyl groups capable of donating hydrogen atoms or electrons to neutralize free radicals [59,60]. Meanwhile, AgNPs, although classically recognized for their antimicrobial action, also possess inherent reductive characteristics [61,62].

**Figure 8:**
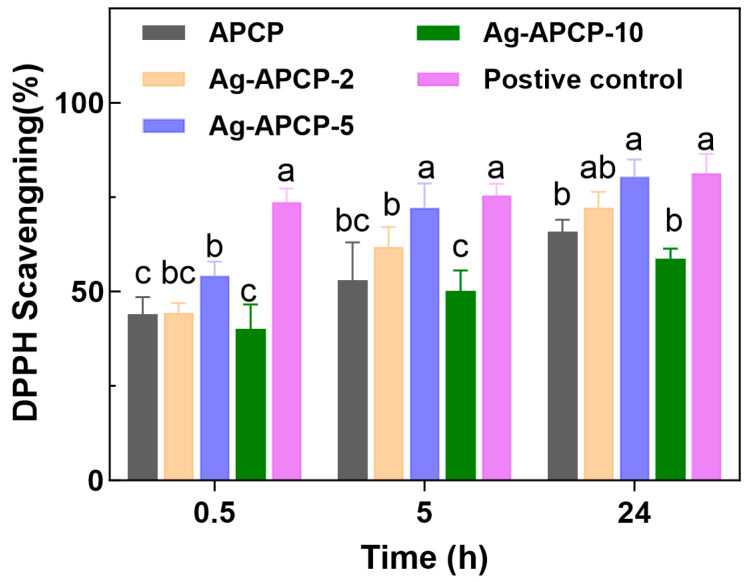
DPPH radical scavenging activity. Different lower-case letters represent significant differences between samples (*p* > 0.05), while the same lowercase letter indicates no significant difference (*p* > 0.05).

To comprehensively evaluate the antioxidant performance of Ag-APCP hydrogels, a DPPH radical scavenging assay was conducted using samples containing 2 wt% APCP and varying concentrations of AgNO_3_ (2, 5, and 10 mmol/L). As shown in Figure 8, all hydrogels exhibited time-dependent enhancement in DPPH radical scavenging activity over the 0.5–24 h range. After 24 h of incubation, the scavenging rates reached 65.13% (APCP), 72.26% (Ag-APCP-2), 78.94% (Ag-APCP-5), and 58.28% (Ag-APCP-10), respectively. Comparative analysis with the blank hydrogel group revealed a positive correlation between AgNO_3_ concentration (2–5 mmol/L) and DPPH scavenging capacity. However, at a higher AgNO_3_ concentration (10 mmol/L), the antioxidant performance of Ag-APCP-10 declined notably. This may be attributed to the excessive consumption of polysaccharide chains during the reduction of Ag^+^ into AgNPs, thereby reducing the number of free hydroxyl or aldehyde groups available for radical scavenging. Similar inhibitory effects have been observed in other polysaccharide-metal hybrid systems, where excessive crosslinking or metal overloading disrupts biofunctionality [63]. Therefore, 5 mmol/L AgNO_3_ concentration was determined to be optimal for fabricating hydrogels with balanced antioxidant performance.

### 2.8. Antibacterial Activity

**Figure 9:**
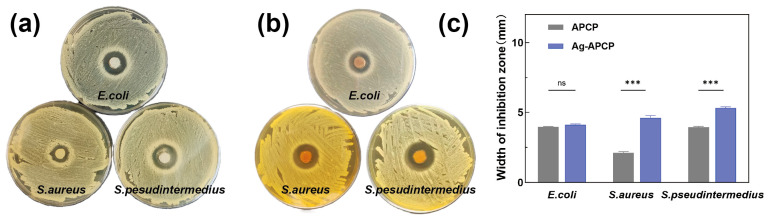
Evaluation of antimicrobial capacity of APCP hydrogels and Ag-APCP hydrogels. Bacteriostatic rings of APCP (**a**) and Ag-APCP (**b**) hydrogels against *E. coli*, *S. aureus*, and *S. pseudintermedius*. Range of bacteriostatic rings (**c**). *** indicates *p* < 0.001, and ns indicates no significant difference.

The inhibition zone results of the APCP and Ag-APCP hydrogels against *Escherichia coli*, *Staphylococcus aureus*, and *S. pseudintermedius* were shown in Figure 9. Both APCP and Ag-APCP hydrogels exhibited antibacterial effects against these three bacterial species. Quantitative measurements (Figure 9c) confirmed that the Ag-APCP group demonstrated the most prominent inhibitory effect against *S. pseudintermedius*, with an average zone of 5.31 mm, followed by *S. aureus* (4.61 mm) and *E. coli* (4.12 mm). All values were higher (*p* < 0.001) than those recorded for the corresponding APCP-only group, except in the case of *E. coli*, where the difference was not statistically significant. Interestingly, these results differ from many previous reports on AgNP-loaded hydrogels, which typically exhibit greater inhibitory effects against Gram-negative bacteria, particularly *E. coli*, due to their thinner peptidoglycan layer and increased membrane permeability to Ag^+^ [64,65,66]. In contrast, in the present study, the strongest antibacterial response was observed against the Gram-positive bacterium *S. pseudintermedius*. This deviation suggests a potential synergistic antibacterial effect between the APCP matrix and AgNPs, possibly through enhanced adhesion or local concentration effects that preferentially affect Gram-positive bacterial membranes.

*S. pseudintermedius* is a pathogen of growing veterinary and zoonotic concern, particularly due to its ability to cause a broad spectrum of infections in canines, especially involving the skin and soft tissues [67,68]. The clinical profile of this organism in canines mirrors that of S. aureus in human populations [68]. Reports have suggested that *S. pseudintermedius* is transmitted via animals to owners and veterinarians [69,70,71,72]. While this zoonotic pathogen is underrecognized in clinical diagnostics, the emerging multidrug-resistant strains amplify zoonotic transmission risks [73,74].

This result contributes a new insight into silver-based antibacterial systems by demonstrating that the APCP-based hydrogel shows unexpectedly higher efficacy against a Gram-positive veterinary pathogen, challenging the typical Gram-negative bias of AgNP sensitivity. This underscores the potential of APCP as a reducing agent and delivery matrix for the development of antimicrobial wound dressings or veterinary coatings.

## 3. Conclusions

This study successfully fabricated a single-component hydrogel via pH-mediated self-assembly of APCP. Structural analysis confirmed that APCP consist predominantly of β-(1→3)-D-glucan. The resulting hydrogel (APCPH) exhibited thermal stability and a three-dimensional porous network. Gelation was driven chiefly by synergistic hydrogen bonding and hydrophobic interactions. This APCPH may potentially facilitate the reduction of Ag^+^ to AgNPs, leading to the formation of Ag–APCP composite hydrogels. While AgNP incorporation had minimal effect on swelling and water-holding capacity, excessive silver loading diminished antioxidant activity. Both the APCP and Ag-APCP hydrogels displayed potent antibacterial activity against *Escherichia coli*, *Staphylococcus aureus*, and *Staphylococcus pseudintermedius*. These findings suggest that a novel strategy for the valorization of alkali-soluble herbal residues, such as APCP, which are typically discarded in traditional extraction processes. Moreover, this work offers direct structural and functional evidence that β-(1→3)-D-glucans possess intrinsic gelation capability, supporting their further exploration as bio-based gel-forming materials.

Nonetheless, this study has certain limitations. The green synthesis of AgNPs was carried out without systematic optimization. In addition, the in vivo biocompatibility and long-term stability of the Ag–APCP hydrogels have yet to be evaluated. To address these limitations, future research will systematically optimize the green synthesis parameters of Ag–APCP hydrogels by adjusting Ag^+^ concentration, pH, temperature, and reaction time to improve nanoparticle stability and enhance functional performance. The antibacterial activity will also be further investigated, particularly against *Staphylococcus pseudintermedius*, to support their potential application in nanomedical and antimicrobial dressings.

## 4. Materials and Methods

### 4.1. Materials and Reagents

The dried sclerotia specimens of P. cocos originated from Yingshan County, Hubei Province and were purchased from Tongrentang Co., Ltd. (Beijing, China). Hydrochloric acid (HCl, 37%), sodium dodecyl sulfate (SDS, 98%), urea (99.5%), and 1,1-diphenyl-2-picrylhydrazyl radical (DPPH, 98%) were purchased from Beijing Solarbio Science & Technology Co., Ltd. (Beijing, China). AgNO_3_ (99.8%) was provided by Sinopharm Chemical Reagent Co., Ltd., Shanghai, China. Ethanol and FT-IR-grade KBr were obtained from Aladdin Biochemical Technology Co. (Shanghai, China). The standard strains of *Escherichia coli* (ATCC 25922), *Staphylococcus aureus* (ATCC 29213), and *Staphylococcus pseudintermedius* (ATCC 49444) were provided by the Clinical Internal Veterinary Medicine laboratory. All remaining chemical reagents conformed to analytical-grade specifications and were supplied by Sinopharm Co., Ltd.

### 4.2. Extraction and Structural Characterization Analysis of Alkali-Soluble Polysaccharides from Poria cocos

We extracted and purified alkali-soluble polysaccharides (APCP) with slight modifications to the methods reported by Li and Wang et al. [75,76]. Briefly, dried *Poria cocos* powder was refluxed twice with 95% ethanol (1:15, *w*/*v*) for 1.5 h each to remove lipophilic compounds. The residue was dissolved in 0.5 M NaOH solution (1:15, *w*/*v*) and stirred continuously at 700 rpm and 25 °C for 2 h. The resulting suspension was centrifuged at 3000 rpm for 15 min at 25 °C to separate the insoluble solids. The supernatant was then slowly neutralized to pH 7.0 using 20% HCl under gentle stirring and allowed to stand at room temperature for 1 h to promote precipitation. The precipitate was collected by centrifugation (3000 rpm, 15 min), washed three times with distilled water, and lyophilized. After lyophilization, the APCP was obtained and stored in a dry, sealed container at room temperature for subsequent experiments.

To analyze the monosaccharide profile of the APCP, samples were separated by high-performance liquid chromatography (HPLC) (U3000, Thermo Scientific, Waltham, MA, USA) equipped with a ZORBAX Eclipse XDB-C18 column. UV detection was performed at 250 nm. The molecular weight distribution of APCP was determined by high-performance gel permeation chromatography (HPGPC) using a Waters E2695 system (Milford, MA, USA) with columns (300 × 8 mm) and 0.05 M NaCl solution as the mobile phase at a flow rate of 0.65 mL/min. Fourier transform infrared spectroscopy (FT-IR) data were recorded using a Nicolet 6700 Fourier (Thermo Scientific, Waltham, MA, USA) transform infrared spectrophotometer in the range of 4000–400 cm^−1^ to identify functional groups and chemical bonds. Glycosidic linkages of the APCP were further characterized by gas chromatography–mass spectrometry (GC-MS; Agilent 7890A–5977B, Santa Clara, CA, USA) following methylation, hydrolysis, and acetylation, using an HP-5MS column (30 m × 0.25 mm × 0.25 μm). Nuclear magnetic resonance (NMR) spectra, including ^1^H NMR, ^13^C NMR, DEPT135, 1D NOESY, ^1^H–^1^H COSY, HSQC, and HMBC, were recorded on a Bruker AVANCE HD III 600 MHz spectrometer (Bruker, Bremen, Germany) at 25 °C. Detailed experimental protocols and conditions are provided in the Supplementary Materials.

### 4.3. Preparation of APCP Hydrogel

To prepare the APCP hydrogel, lyophilized APCP was added to 0.5 M NaOH solution at a concentration of 1–3 wt%, based on preliminary solubility and gelation screening. The suspension was stirred magnetically at 37 °C for 30 min to ensure complete dissolution under alkaline conditions, which facilitate polysaccharide chain relaxation and dispersion. Subsequently, 20% HCl was added dropwise under continuous stirring to adjust the solution to the target pH (4.0, 6.0, 7.0, or 8.0). The resulting mixture was gently stirred at 37 °C and then allowed to stand at room temperature to stabilize the hydrogel network. Hydrogels with varying APCP concentrations (1, 2, and 3 wt%) and pH values were prepared using the same procedure for comparative analysis of gelation behavior and physical properties.

### 4.4. Rheological Properties

Rheological characterization of the APCPH was performed using a modular rheometer (MCR 102, Anton Par, Graz, Austria) with parallel plate geometry (50 mm diameter, 1 mm gap) at 25 °C. After loading the hydrogel sample between the plates, a thin layer of silicone oil was applied around the edge to prevent solvent evaporation during measurement. Viscoelastic parameters, including storage modulus (G′) and loss modulus (G”), were recorded.

To evaluate the viscoelastic behavior of the hydrogels, both dynamic strain sweep and frequency sweep tests were conducted [77]. A strain sweep (0.1–100%) was first performed at a fixed frequency of 1 Hz to identify the linear viscoelastic region (LVR), ensuring that subsequent measurements remained within the range where the material structure was not disrupted. Based on the results, a fixed strain amplitude of 1.0%—within the LVR—was selected for the frequency sweep analysis (0.1–100 Hz), which assessed the dependence of G′ and G″ on oscillation frequency. This approach allowed reliable comparison of gel strength and structural stability under small deformations.

### 4.5. Characterization of APCP Hydrogel

#### 4.5.1. Scanning Electron Microscope (SEM) Observations

The microstructure of the APCPH was observed using a scanning electron microscope (Helios 5 CX, Thermo Scientific, Waltham, MA, USA) at 15 kV. APCPH samples were cryofixed at −80 °C and stabilized for 24 h prior to lyophilization to preserve structural integrity. Cross-sectional morphology was obtained by brittle fracture, and samples were sputter-coated with a thin layer of gold to improve imaging resolution.

#### 4.5.2. Differential Scanning Calorimetry (DSC) Analysis

The thermal properties of the APCPH were analyzed by DSC using a differential scanning calorimeter (Netzsch STA 449 F5/F3, Selb, Germany) under nitrogen protection. With reference calibration using an empty container, 5 mg of dried hydrogel was sealed in a standard aluminum crucible and scanned from 20 to 200 °C at a heating rate of 10 °C/min.

### 4.6. Determination of Molecular Forces

According to the method reported by Wang et al. [78,79], gelation mechanisms were investigated using dissociation agents (urea, SDS, and NaCl). Each agent was dissolved in 2 wt% APCP alkaline solution and the pH was adjusted to 6.0 using 20% HCl. The final concentrations of SDS and urea were 0.01, 0.025, 0.04, and 0.2 wt%, while those of NaCl were 0.05, 0.15, 0.20, and 0.30 M. The mixtures were stored at 25 °C for 12 h. APCPH without dissociation agents served as the control. Sol–gel transition states were recorded to construct state diagrams. Additionally, the effect of NaCl on the rheological behavior of the APCP hydrogel was assessed as described in Section 2.4.

### 4.7. Synthesis and Morphological Characterization of AgNPs

Based on Section 4.3, the optimized synthesis of AgNPs was performed. AgNO_3_ solutions were prepared and stored in the dark. Lyophilized APCP was dissolved in 0.5 M NaOH to obtain 2 wt% solutions, which were then adjusted to pH 7.0 using 20% HCl. AgNO_3_ was subsequently added to achieve final concentrations of 2, 5, and 10 mM, yielding Ag-APCP-2, Ag-APCP-5, and Ag-APCP-10 hydrogels, respectively. The mixtures were incubated at 50 °C in the dark for 60 min and then stored at 4 °C for 12 h prior to further use.

The morphology of AgNPs was observed using transmission electron microscopy (TEM; Hitachi H7600, Tokyo, Japan). The Ag-APCP-5 hydrogel was dispersed in deionized water, and a small aliquot of the suspension was dropped onto a copper grid. After drying for 24 h, the sample was examined at 90 kV.

### 4.8. Swelling Ratios and Water-Holding Capacities of APCP Hydrogels and Ag-APCP Hydrogels

#### 4.8.1. Swelling Properties

Lyophilized hydrogels underwent hydration equilibrium in deionized water at room temperature. Surface water was gently removed and sample weight was measured at predetermined time points until constant mass was observed. Measurements were conducted in triplicate using independently prepared hydrogels. The swelling ratio was quantified according to the formula

Swelling ratio (100%) = (W_t_ − W_0_)/W_0_ × 100%

where W_0_ and Wₜ denote the original mass of lyophilized hydrogel specimens and their swollen-state mass measured at defined temporal intervals, respectively.

#### 4.8.2. Water Holding Capacity

Freshly prepared hydrogels were stood at 25 °C for 2 h, followed by centrifugation at 5000 rpm for 15 min. Surface water was removed with dry filter paper. Experiments were performed in triplicate. The water-holding capacity (WHC) was calculated using the formula

WHC (%) = W_t_/W_0_ × 100%

where W_0_ and W_t_ represent the weight of the initial weight of the hydrogel and the weight of the hydrogel after water removal, respectively.

### 4.9. Antioxidant Activities of APCP Hydrogels and Ag-APCP Hydrogels

The antioxidant properties of the hydrogels were investigated using DPPH radical scavenging assay. Each hydrogel sample was added to 3 mL of 100 μM ethanolic DPPH solution in the dark at room temperature. A 0.5 mM l-ascorbic acid solution was used as the positive control. Absorbance measurements were conducted at 517 nm using a UV–vis spectrophotometer (UV-2600, Shimadzu, Kyoto, Japan) at designated time points. All measurements were repeated three times. Radical scavenging efficiency (%) was determined through the equation

DPPH Scavenging (%) = [(A_0_ − A_s_)/Ab] × 100%

where A_0_ is the absorbance of the control group and A_s_ is the absorbance of the sample.

### 4.10. Antibacterial Activity of APCP Hydrogels and Ag-APCP Hydrogels

The antibacterial activity of the APCPH and Ag-APCP-5 hydrogels against pathogens (*Staphylococcus aureus*, *Staphylococcus pseudointermedius*, and *Escherichia coli*) was evaluated using the Kirby–Bauer disk diffusion method. Briefly, 200 μL of bacterial suspension (10^6^ CFU/mL) was evenly spread on BHI agar plates. Hydrogel discs (7 mm diameter and 2 mm thickness) were placed on the agar surface. The plate were inverted and incubated at 37 °C for 24 h. The diameter of the inhibition zone was measured. All experiments were performed in triplicate.

### 4.11. Statistical Analysis

All experiments were conducted in triplicate (*n* = 3), and results are expressed as mean ± standard deviation (SD). Data normality was assessed using the Shapiro–Wilk test. For two-group comparisons, an unpaired *t*-test was used; for comparisons among multiple groups, one-way ANOVA followed by Tukey’s post hoc test was applied. A *p*-value < 0.05 was considered statistically significant. Statistical analyses were performed using GraphPad Prism (v10.1.2; GraphPad Software, San Diego, CA, USA).

## Figures and Tables

**Table 1:** The monosaccharide composition and molecular mass of APCP.

APCP	Man (%)	Glc (%)	Mw (kDa)	Mn (kDa)	Mw/Mn
	0.10%	99.896	314.2	237.4	1.32

**Table 2:** Methylation analysis of the APCP.

Glycosidic Linkage	PMAA	Retention Time (min)	Molar Fraction (%)
t-Glc(p)	1,5-di-O-acetyl-2,3,4,6-tetra-O-methyl glucitol	16.809	12.272
3-Glc(p)	1,4,5-tri-O-acetyl-2,3,6-tri-O-methyl glucitol	19.827	77.497
3,4-Glc(p)	1,3,4,5-tetra-O-acetyl-2,6-di-O-methyl glucitol	22.018	1.244
2,3-Glc(p)	1,2,3,5-tetra-O-acetyl-4,6-di-O-methyl glucitol	22.349	4.018
3,6-Glc(p)	1,3,5,6-tetra-O-acetyl-2,4-di-O-methyl glucitol	23.483	4.970

**Table 3:** Chemical shift assignments of the APCP.

Residue		Chemical Shift (ppm)							
			1	2	3	4	5	6a	6b
G_3_	→3)-β-D-Glcp-(1→	H	4.71	3.47	3.7	3.43	3.43	3.66	3.84
		C	102.46	73.08	84.1	67.88	75.5	60.45	
G_36_	→3,6)-β-D-Glcp-(1→	H	4.72	3.46	3.71	3.45	3.44	--	--
		C	102.37	73.09	84.12	67.97	75.44	--	
G_t_	β-D-Glcp-(1→	H	4.67	3.26	3.45	3.33	3.39	3.65	3.84
		C	102.52	73.28	75.55	69.53	75.5	60.41	
R_α_	→3)-α-D-Glcp	H	5.14	3.62	3.83	3.40	3.78	3.74	3.79
		C	91.79	70.91	82.02	69.44	71.00	60.35	
R_β_	→3)-β-D-Glcp	H	4.59	3.34	3.46	3.44	3.32	3.64	3.80
		C	95.65	73.80	83.78	67.92	75.73	60.42	

## Data Availability

The data presented in this study are available on request from the corresponding author.

## Supplementary Materials

The following supporting information can be downloaded at: https://www.mdpi.com/article/10.3390/gels11060387/s1, Figure S1: The ^1^D NOESY, DEPT 135 and HMBC, NOESY spectra of APCP; Figure S2: Macroscopic diagram of hydrogel and gelation time under different conditions; Figure S3: Effects of SDS, urea and NaCl addition on APCP hydrogels; Figure S4: Effects of NaCl on G′ and G′′ of APCP hydrogels.
