# Supplementary material for: Structural and Gelation Characteristics of Alkali-Soluble β-Glucan from Poria cocos
Source: Gels. 2025 May 24;11(6):387. doi: 10.3390/gels11060387 (PMC12191588; doi:10.3390/gels11060387)
Supplement: Supplementary file 1 [file gels-11-00387-s001.zip › gels-3602175-supplementary.pdf]

Supplementary

Alkali-soluble polysaccharide from Poria cocos：Structural characteristics、gelation properties and potential green synthesis of silver nanoparticles

Zhixing Li[1], Chenglei Sun[2], Fan Wang[1], Zhaofei Xia[1,*]

*Correspondence: zhaofeixiacau@126.com

Affiliations

1 College of Veterinary Medicine, China Agricultural University, Beijing 100193, China.

2 College of Agricultural and Life Sciences, The University of Tokyo, Tokyo 113-8654, Japan.

Materials and Methods

Methods

1. Monosaccharide Composition Analysis

Monosaccharide profiling of APCP was performed using a Thermo Scientific UltiMate 3000 HPLC system (USA) equipped with a ZORBAX Eclipse XDB-C18 column. Prior to analysis, APCP was hydrolyzed with 2 M trifluoroacetic acid (TFA) at 121 °C for 2 h. The hydrolysates were derivatized with 1-phenyl-3-methyl-5-pyrazolone (PMP) and separated at 30 °C with UV detection at 250 nm. The mobile phase consisted of 0.1 M phosphate buffer (pH 6.8) and acetonitrile (83:17, v/v), at a flow rate of 1.0 mL/min. The monosaccharide composition of APCP was identified by comparing its retention times with those of standard sugars analyzed under identical chromatographic conditions.

2. Molecular Weight Distribution

The weight-average molecular weight (Mw) of APCP was determined using high-performance gel permeation chromatography (HPGPC) using a Waters E2695 system with columns (Ohpak, 300 × 8 mm). APCP was dissolved in 0.05 M NaCL and injected into a series of columns (300 mm × 8 mm). The column temperature

was maintained at 40 °C, with a flow rate of 0.65 mL/min and injection volume of 30 μL.

3. Fourier transform infrared（FT-IR） Spectroscopy

FT-IR spectra were recorded using a Nicolet 6700 spectrophotometer (Thermo Fisher Scientific, USA) over the range of 4000–400 cm^{-1} with a resolution of 4 cm^{-1}. Lyophilized APCP powder (1–2 mg) was mixed with KBr and pressed into pellets prior to analysis.

4. Methylation and Linkage Analysis

Methylation of APCP was carried out using a modified Ciucanu–Kerek method. Briefly, 1–3 mg of APCP was dissolved in 500 μL DMSO, followed by the addition of 1 mg NaOH. After 30 min of incubation at room temperature, 50 μL methyl iodide was added, and the reaction proceeded for 1 h. The mixture was extracted with water and dichloromethane (DCM), and the organic phase was washed three times with water and evaporated to dryness. The residue was hydrolyzed in 100 μL 2 M trifluoroacetic acid (TFA) at 121 °C for 90 min, dried at 30 °C, and reduced using 50 μL 2 M ammonia and 50 μL 1 M $NaBD_4$ for 2.5 h. The reaction was terminated with 20 μL acetic acid, followed by methanol washing and nitrogen drying. Acetylation was performed with 250 μL acetic anhydride at 100 °C for 2.5 h. After quenching with water, the product was extracted with 500 μL DCM, washed three times with water, and the organic phase was analyzed by GC–MS.

GC–MS analysis was conducted on an Agilent 7890A gas chromatograph coupled with a 5977B mass spectrometer (Agilent Technologies, USA), equipped with a DB-5MS capillary column (30 m × 0.25 mm × 0.25 μm). The injection was performed in split mode (10:1) with a volume of 1 μL at 260 °C. The oven program

was: 50 °C (1 min), ramped to 130 °C at 50 °C/min, then to 230 °C at 3 °C/min, held for 2 min. Helium was used as the carrier gas at 1.0 mL/min. Mass spectra were recorded in EI mode (70 eV) in full-scan range (m/z 30–600), with ion source and quadrupole temperatures set at 230 °C and 150 °C, respectively.

5. Nuclear Magnetic Resonance (NMR) Spectroscopy

The structural features of APCP were characterized by one-dimensional (1D) and two-dimensional (2D) NMR spectroscopy. Freeze-dried APCP was dissolved in 0.5 mL of D_2O and analyzed using a Bruker AVANCE III 600 MHz NMR spectrometer (Bruker BioSpin, Germany) at 25 °C. The experiments included ^1H NMR, ^{13}C NMR, DEPT-135, and 2D techniques such as COSY, HSQC, HMBC, and NOESY.

Chemical shifts were referenced to residual HDO at δ_H = 4.70 ppm and to the internal TMS carbon signal at δ_C = 0.00 ppm. All spectra were acquired using standard Bruker pulse sequences.

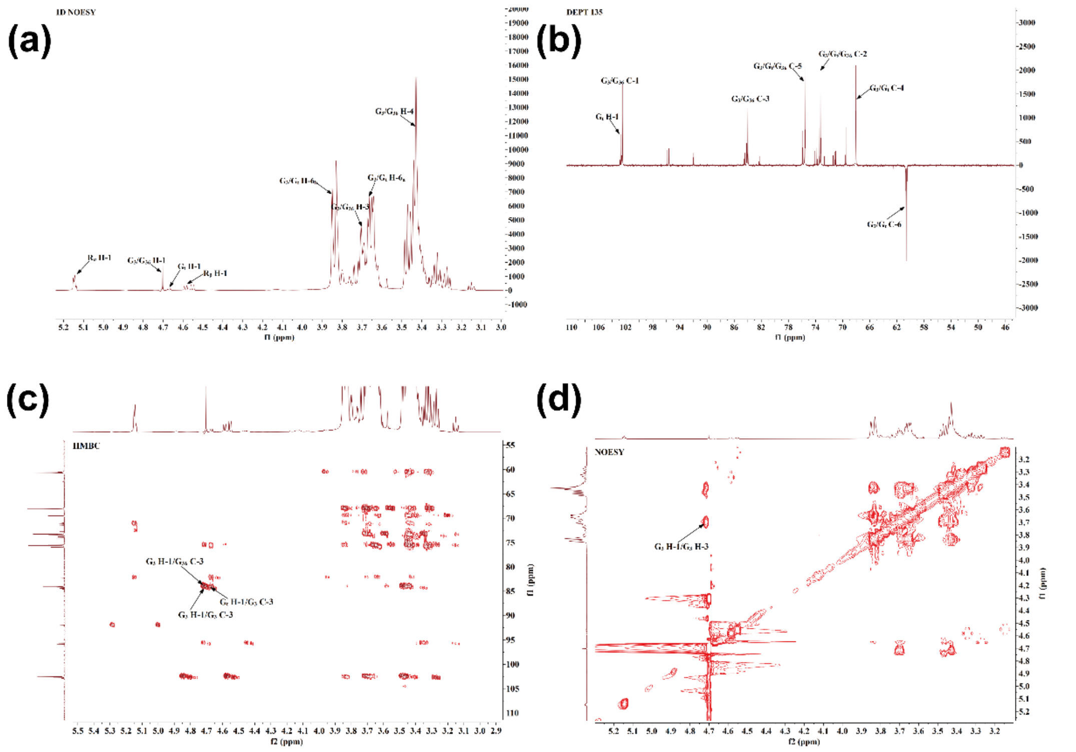

Figure S1. The ^{1}D NOESY, DEPT 135 and HMBC, NOESY spectra of APCP (a-d).

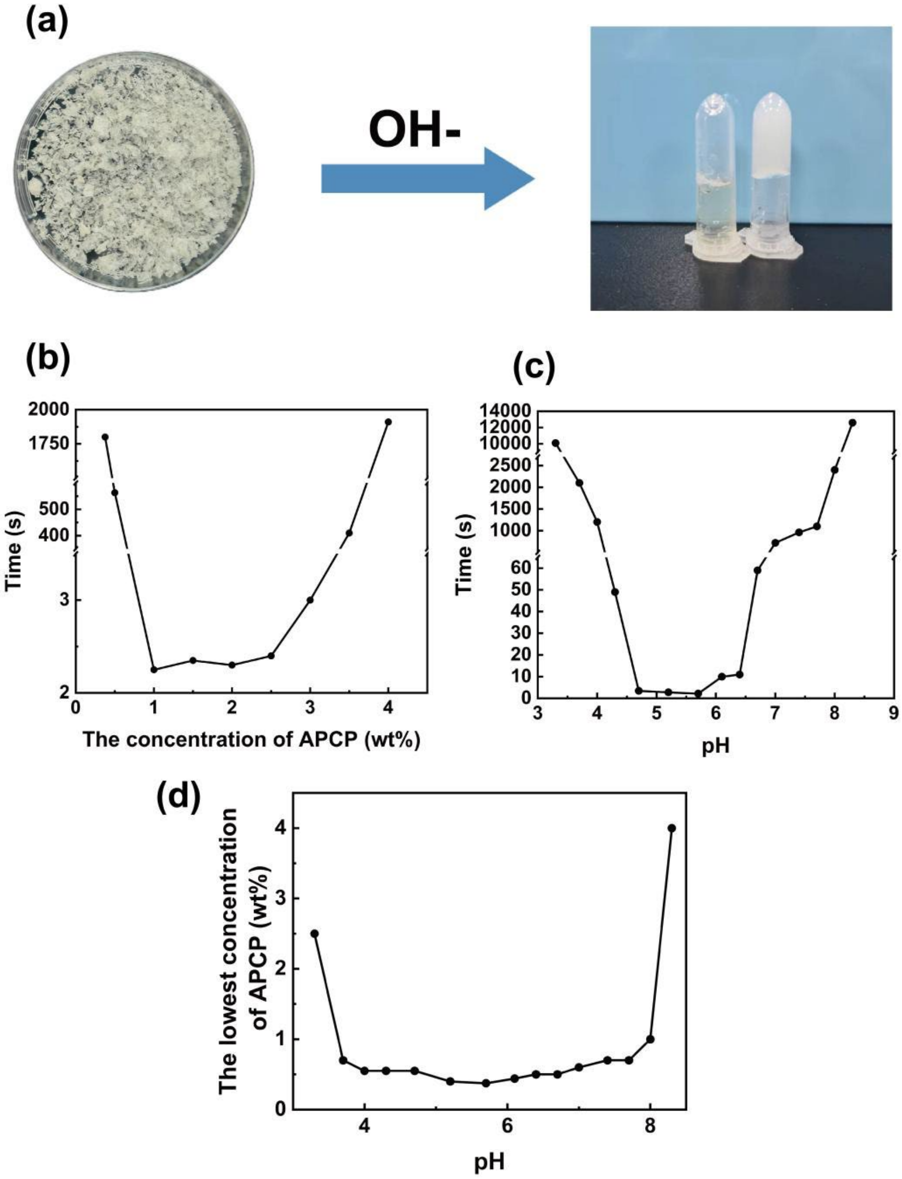

Figure S2. Macroscopic diagram of hydrogel and gelation time under different conditions. (a) Macroscopic schematic diagram of freeze-dried APCP and APCP hydrogel; (b) Gel time at different APCP concentrations; (c) Gel time at different pH; (d) Lowest gel concentration at different pH

SDS(wt%)	0	0.01	0.025	0.04	0.2
State	✓	✓	✓	※	※
Urea(wt%)	0	0.01	0.025	0.04	0.2
State	✓	✓	※	※	×
NaCl(M)	0	0.05	0.15	0.2	0.3
State	✓	✓	✓	✓	✓

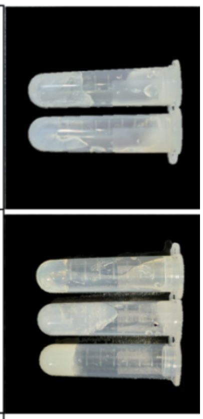

Figure S3. Effects of SDS, urea and NaCl addition onAPCP hydrogels：(✓) represents the gel state; (※) represents the semi-gel state; (×) represents the liquid state.

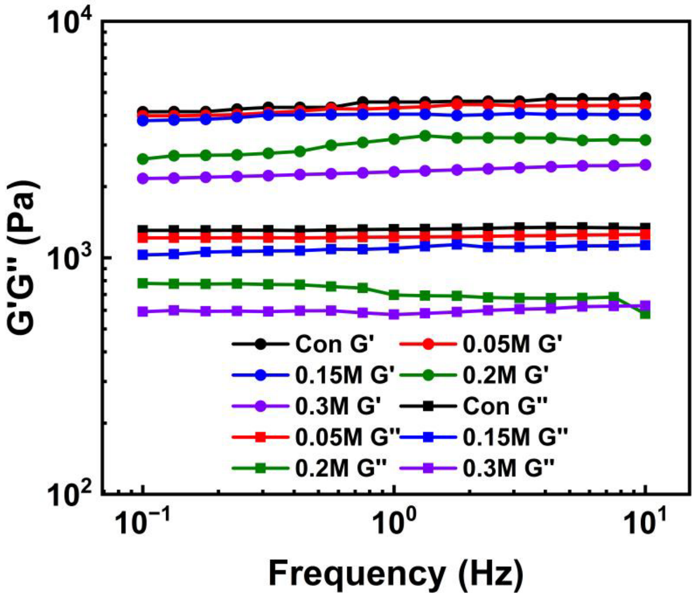

Figure S4. Effects of NaCl on G′ and G″ of APCP hydrogels
